# The Impact of Simulated Bruxism Forces and Surface Aging Treatments on Two Dental Nano-Biocomposites—A Radiographic and Tomographic Analysis

**DOI:** 10.3390/medicina59020360

**Published:** 2023-02-14

**Authors:** Amelia Anita Boitor, Elena Bianca Varvară, Corina Mirela Prodan, Sorina Sava, Diana Dudea, Adriana Objelean

**Affiliations:** 1 Department of Dental Propaedeutics and Esthetics, Faculty of Dental Medicine, University of Medicine and Pharmacy “Iuliu Hatieganu”, 400006 Cluj-Napoca, Romania; 2 Department of Dental Materials and Ergonomics, Faculty of Dental Medicine, University of Medicine and Pharmacy “Iuliu Hatieganu”, 400083 Cluj-Napoca, Romania

**Keywords:** CBCT, nanohybrid biocomposites, masticatory parafunction, surface aging treatment, dental bleaching, digital X-ray, two-body wear simulation, bruxism, imaging in dentistry

## Abstract

*Background and Objectives*: Nowadays, indication of composite materials for various clinical situations has increased significantly. However, in the oral environment, these biomaterials are subjected (abnormal occlusal forces, external bleaching, consumption of carbonated beverages, etc.) to changes in their functional and mechanical behavior when indicated primarily for patients with masticatory habits. The study aimed to recreate in our lab one of the most common situations nowadays—in-office activity of a young patient suffering from specific parafunctional occlusal stress (bruxism) who consumes acidic beverages and is using at-home dental bleaching. *Materials and Methods*: Sixty standardized class II cavities were restored with two nanohybrid biocomposite materials (Filtek Z550, 3M ESPE, and Evetric, Ivoclar Vivadent); the restored teeth were immersed in sports drinks and carbonated beverages and exposed to an at-home teeth bleaching agent. The samples were subjected to parafunctional mechanical loads using a dual-axis chewing simulator. A grading evaluation system was conducted to assess the defects of the restorations using different examination devices: a CBCT, a high-resolution digital camera, and periapical X-rays. *Results*: Before mechanical loading, the CBCT analysis revealed substantially fewer interfacial defects between the two resin-based composites (*p* > 0.05), whereas, after bruxism forces simulation, significantly more defects were identified (*p* < 0.05). Qualitative examination of the restorations showed more occlusal defects for the Evetric than the other nanohybrid composite. *Conclusions*: There were different behaviors observed regarding the studied nanocomposites when simulation of parafunctional masticatory forces was associated with aging treatments.

## 1. Introduction

It is known that the most resistant material that withstands the oral cavity environment is the natural tooth, with its biological tissues, enamel, and dentine [1]. However, due to possible tooth diseases or traumas, these naturally engineered tissues may be damaged by carious lesions, resulting in tooth decay and defects that need to be reconstructed with oral biomaterials. These materials must be biocompatible, with optimal physical, mechanical, chemical, and aesthetic properties. One of the most often indicated groups of restorative materials is resin composite, which has shown an acceptable survival rate in clinical and in vitro studies [2].

During recent decades, research and development of resin-based composites have generated different subcategories of restorative materials that include composites containing nano-sized filler particles [3,4,5,6]. Nano-filled composites have nanometric-sized particles, while nanohybrid ones contain finely ground glass fillers and nano-fillers in a pre-polymerized filler form [7,8]. Some professionals claim that the newly introduced materials [7] offer reduced polymerization contraction, enhanced mechanical properties, and improved aesthetics [7,8,9].

Aesthetics has always been an important topic for patients. Thus, for over two decades, bleaching treatments, especially those carried on at home, under a doctor’s supervision, are often treatments of choice for improving dental color in adolescents and young adults, particularly those interested in their body image [8,9,10,11]. This group of patients is known to prioritize body aesthetics and practice sports regularly. Along with cutting-edge new technologies in sports coming into play, there are also various sports drinks, energy drinks, or soft drinks containing other sugary and mineral compounds [10].

It was reported in the literature that oral biofilm might induce surface changes in dental restorative materials, leading to their chemical degradation and thus a higher chance for the dental fillings to develop marginal percolation and other defects [12]. It was also reported that chewing gum might lead to leaching of chemical compounds of resin-based composites [13]. It is well known that any change in the architecture of the oral cavity may interfere with the whole oral equilibrium, disturbing it at different levels (e.g., muscles, teeth, TMJ) [8,9]. Consequently, parafunctional occlusal habits that generate increased forces on teeth are frequently encountered and reported to highly affect tooth-adhesive interface and tooth wear strength [1,8,9,14].

In this established environment, dramatic oral changes were observed due to low-pH drink consumption and at-home dental bleaching or other whitening pastes. This problem could be clinically translated with dental erosions and surface and interfacial failure of different dental fillings [12,13,15,16,17,18,19,20]. 

Micro-computer tomography is a non-destructive X-ray 3D image analyzer used mainly in laboratory investigations. Digital radiography is the most often clinically indicated imagistic analysis used by dental practitioners. This radiographic method uses 2D images, but new systems or devices can analyze a broader range of oral environment changes. One of these systems is cone-beam computed tomography (CBCT), which offers a three-dimensional view of oral tissues for different clinical indications (such as caries detection, TMJ disorders, or bone density) [21,22]. A few years ago, CBCT was proposed as a 3D image analyzer for in vitro studies [22,23,24]. 

The literature did not thoroughly analyze the consequences of combined patients’ high aesthetic demands, sugar-added carbonated beverages, and parafunctional occlusal habits. Based on the research data on this subject, the present in vitro study aimed to assess behavior of two direct resin-based nanocomposites subjected to two surface aging treatments (external bleach and acid beverages) combined with simulated occlusal parafunctional forces.

The null hypothesis was that the two restorative nano-biocomposites had very similar mechanical, aesthetic, and functional behaviors regardless of type of surface aging and higher impact loads applied. 

## 2. Materials and Methods

The study protocol was approved by the Research Ethics and Methodology Department, University of Medicine and Pharmacy “Iuliu Hatieganu”, Cluj-Napoca, Romania (247/30.06.2021).

For this investigation, the sample size was determined based on a previous pilot study, for which the effect size was 1, power (1-β) of 0.8, and the level of significance = 0.05. The data were analyzed using a *t*-test family for matched pairs, with G*power software version 3.1.9.7 (Kiel University Software, Germany) for Windows software. The optimal sample size was calculated as up to 10 dental cavities based on the abovementioned assumptions.

Before the experimental test, the teeth were checked for cracks, fractures, or other surface defects using dental loupes (3× magnification) and a sharp explorer. Then, the teeth were cleaned, and any soft tissue was removed using an ultrasound scaler (U600 LED, Woodpecker Medical Instruments Co. Ltd., Guilin, China). After the cleaning procedure, the teeth were kept in 1% Chloramine T, and, after a thorough rinse, they were stored in distilled water at 4 °C.

Sixty standardized class II cavities (mesio-occlusal, disto-occlusal) were prepared on intact human premolars extracted for periodontal or orthodontic reasons upon the patient’s consent. Two proximal cavities were prepared for each tooth with the following dimensions: 3 mm occlusal depth, 4 mm in buccal-lingual width, 2 mm proximal depth, 4 mm width at the cervical area; all the proximal cavities had the gingival margin placed at 1 mm above the cementoenamel junction (CEJ). The teeth were randomly divided into two groups (n = 30 cavities/material) and restored using the following nanohybrid resin-based composites: Group 1, Evetric, Ivoclar Vivadent (Gr 1 EV); Group 2, Filtek Z550, 3M ESPE (Gr 2 FZ). A self-etch universal adhesive (Opti-bond XTR Universal Self-Etch, Kerr Corp, USA) was applied before the respective restorative composite (). The composites were applied based on the zig-zag technique (2 mm/layer/material). For polymerization of each increment (40 s/layer), we used a 2nd generation LED light-curing lamp (SDI, Radi-plus, light intensity = 1500 mW/cm2; wavelength range = 440–480 nm). The tip of the curing lamp was placed in direct contact with the dental wall where the material increment was placed. Then, the restorations were finished and polished using oval carbide burs (SS White, T&F, Carbide burs, FG, 7406), Sof-Lex abrasive disk (3M ESPE), Occlubrush impregnated polishers (Kerr Co.), and Super Polish Paste (Kerr Co.).

The teeth were immersed twice a day for 28 days in a sports drink ((Gatorade Red Orange with a pH of 3.2) and then in a carbonated beverage (Coca-Cola, pH = 2.4)) [10]. The pH of the two beverages was tested using a pH meter HI-98103 (Hanna Instruments) three times for each. After immersion in each acid beverage, the teeth were thoroughly cleaned and rinsed. Then, they were subjected to external home bleaching treatment (Natural White 5 min Whitening) for 14 days, 5 min/day, according to the manufacturer’s recommendations [25]. After external bleaching procedures, the samples were cleaned and rinsed with distilled water.

For high-impact occlusal forces simulation, a dual-axis chewing simulator was used (CS 4.2, SD Mechatronik, Germany). Before the simulation, the roots of the restored teeth were wax-sealed apically, covered with a type 3 polyvinyl siloxane (PVS), and then embedded in self-cured acrylic resin according to a previously published article [14]. While subjected to mechanical loading and surface treatments, the restored teeth were immersed in artificial saliva.

In the chewing simulator device, the restored teeth were placed side by side in the test chambers so that the stylus simultaneously touched the contact point between two opposed adjacent restorations. Thus, half of the samples from each nanocomposite were subjected to mechanical loads and surface aging treatments (ML + ST, n = 15 samples). Half of them were only subjected to surface treatments (ST, n = 15 sample restorations). The following parameters were used for parafunctional mechanical loading: 125,000 cycles at 7 kgf (70N) per stylus at 1.6 Hz frequency with lateral travel of ±3 mm. 

**Figure 1:**
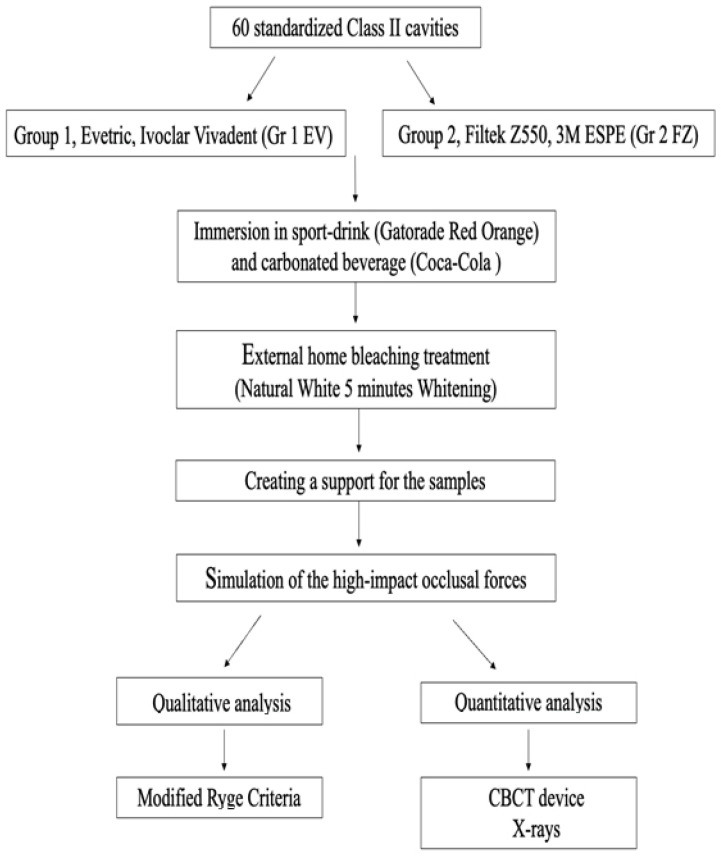
The workflow of the protocol used in the study.

The description of the setup protocol is shown in Figure 1.

The samples were covered with two layers of nail polish with 1 mm preservation around the margins of the restorations; then, the restored teeth were immersed in 50 wt% of silver nitrate solution. After a thorough rinse, the teeth were immersed in a photo-developer for eighth (Dental X-ray Developer, Kodak Co, Rochester, NY, USA) and analyzed with a digital X-ray and CBCT device.

Qualitative analysis was accomplished by one observer according to macro-morphological characteristics based on Modified Clinical parameters criteria [26,27] using dental loupes (3× magnification) and a high-resolution digital camera.

Using modified clinical parameters criteria, the restored teeth were observed and evaluated for the following characteristics based on a grading system (Gr 0–Gr 2) (Table 1):

1. Color match (CM)
2. Marginal adaptation (MA)
3. Surface roughness (SR)
4. Anatomical form (AF)

Detailed information on the materials used in the study appears in Table 2 and Table 3.

For quantitative assessment of the dental filling defects, the following devices were used: the digital X-rays device and the CBCT device (Planmeca USA Inc, Hoffman Estates, IL, USA) (the thickness of the smallest slice was established at 200 µm).

### Statistical Analysis

For statistical analysis, the samples were evaluated by material, by grade of defects, and by mechanically loaded or not. Shapiro–Wilk test was performed to test the normal distribution of the results. The data were subjected to different non-parametric statistical tests (Kruskal–Wallis, Mann–Whitney U) using IBM SPSS software, version 21.0 (SPSS Inc, Chicago, IL, USA). The Spearman rho test was used to verify any statistical correlation between the extrinsic modified clinical parameters criteria system and the radiographic and tomographic intrinsic grading system. The level of statistical significance was established at *p* < 0.05.

## 3. Results

This study observed the restorations after surface aging treatments (ST) and after the mechanical loading and surface treatments were applied (ML + ST) and evaluated them based on qualitative and quantitative analysis. 

### 3.1. Qualitative Analysis

When using the modified clinical parameters criteria to compare the two tested materials, the statistical analysis revealed the following: 

(1) For color match parameters (CM) of Filtek Z550 and Evetric restorations, the Kruskal–Wallis test showed significant differences among the grades for both aging treatments (ML + ST and ST) (*p* < 0.0000001) (Table 4).

The Mann–Whitney test indicated the following results: for ST and ML + ST aging groups, higher statistically significant differences for Gr 0 compared to Gr 1 and Gr 2 for both tested restorative materials (*p* < 0.05). Less statistically significant color changes were observed for Grade 2 compared with Grade 1 for tested materials and aging treatments (*p* < 0.05).

**Figure 2:**
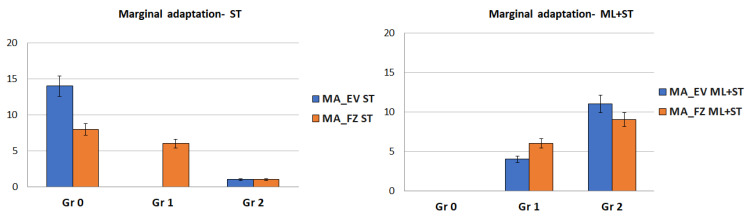
Error bars chart representing the ‘grades’ distribution of the marginal adaptation (MA) parameter.

**Figure 3:**
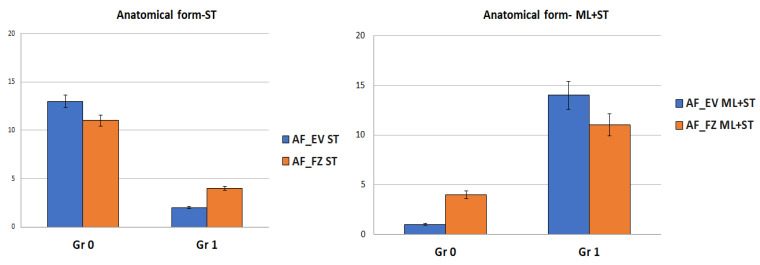
Error bars chart representing the ‘grades’ distribution of the anatomical form (AF) parameter.

(2) When the Kruskal–Wallis test was applied, the marginal adaptation parameter (MA) for both resin-based composite materials had statistically significant differences among the grades for both aging treatment groups (*p* < 0.05). After being subjected to mechanical forces (ML + ST), both materials showed higher statistically significant values for Grade 2 (Figure 2). For the restorations that were only subjected to surface treatments (ST), the Mann–Whitney test showed a higher amount of Evetric samples without marginal adaptation defects (Gr 0) than the Filtek restorations. 

(3) For the surface roughness parameter (SR), the Kruskal–Wallis statistical test revealed significant differences only for the Evetric ST group between the grades (*p* < 0.000001) (Table 5). At the same time, the Mann–Whitney U test showed significantly higher values for Gr 1 than Gr 0 (*p* = 0.001) for the same group. After the Evetric samples were subjected to mechanical loading treatment (ML + ST), a higher number of restorations with Gr 2 were observed (*p* > 0.05). Regarding Filtek Z550 samples, the statistical tests did not show significant differences among the tested groups (ST versus ML + ST) (Table 5).

(4) For the anatomical form parameter (AF), the statistical tests indicated significant differences for both groups of restorations no matter whether they were subjected or not to simulated bruxism forces (*p* < 0.05) (Figure 3).

**Figure 4:**
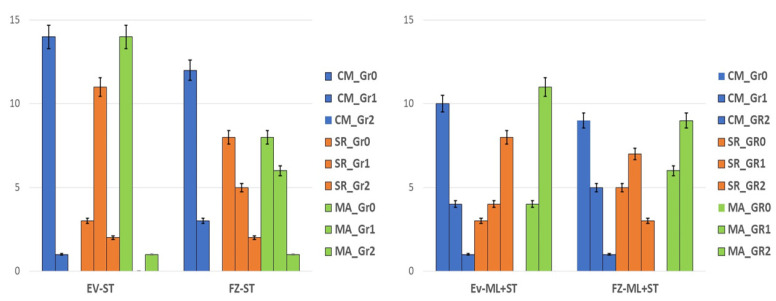
Error bars chart representing the restorative composite materials by applied aging methods among the modified Ryge criteria parameters.

When comparing the restorations by material, the Kruskal–Wallis test did not reveal any statistical difference in terms of color changes between the tested resin composites for both mechanically and non-mechanically loaded groups (ML + ST and ST) (*p* > 0.05). On the other hand, when comparing surface roughness (SR), a smoother surface (Gr 0) was observed for the samples restored with FZ material compared to EV after surface aging treatments (ST) (*p* = 0.021) (Figure 4).

The Kruskal–Wallis test revealed no defects of marginal adaptation for the EV restorations after surface treatments (ST) compared to FZ restorations (*p* < 0.00001). On the other hand, similar marginal adaptation defects were observed for both resin-based biocomposites subjected to ML + ST (*p* > 0.05) (Figure 4). 

### 3.2. Quantitative Analysis

The quantitative evaluation was based on a grading system of the defects that appeared at the interface and occlusal level of the restorations, respectively (Table 6 and Table 7). Importantly, all the restorations were assessed with the help of periapical X-ray and CBCT images.

**Figure 5:**
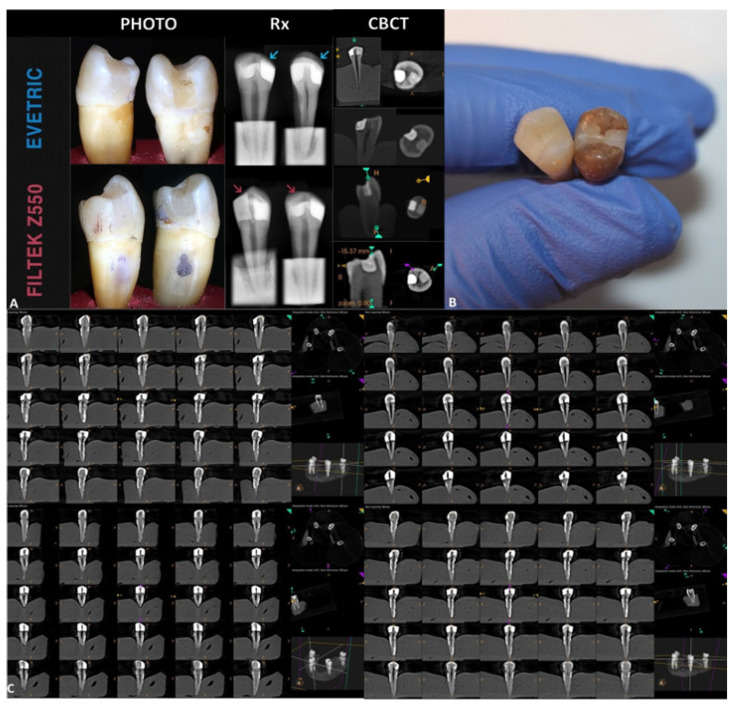
(**A**) Qualitative evaluation of the restorations based on high-resolution photos, digital X-rays, and CBCT images. (**B**) Restored teeth samples before and after immersion in sports drinks and carbonated beverages. (**C**) CBCT images of 200 µm slice evaluating the occlusal and interfacial defects after the bruxism forces simulation.

It was observed that Evetric restorations were more radio-opaque than Filtek Z550 restorations (Figure 5). More cervical defects were observed radiographically for the Evetric nanohybrid material compared with the samples restored with Filtek Z550. When degree of impairment at the level of dental fillings was evaluated based on CBCT images, Filtek Z550 showed similar occlusal defects with Evetric biocomposite (*p* > 0.05). At the same time, for Filtek Z550 composite material, we observed a detachment line at the tooth-restoration interface after high-impact bruxism simulation.

The Kruskal–Wallis test revealed statistically significant differences between grades of impairment for Evetric composite for both situations (with and without high occlusal forces simulation) (*p* = 0.001). For Filtek Z550 material, similar behavior was observed for samples were treated only with sports drink/carbonated juice and bleaching substance (ST) (*p* = 0.56). In contrast, for those that underwent high-impact occlusal force simulation (ML + ST), a statistically significant difference was observed between grades of impairment (*p* = 0.017) (Figure 5A). 

Mann–Whitney U test showed a statistically significantly lower number of non-mechanically loaded restorations (ST) of Filtek Z550 composite material that presented Gr 0 interfacial defects compared with Evetric (*p* = 0.029). For Grade 2, there were not found any statistical differences between samples of materials that were not subjected to parafunctional simulation (ST) (*p* > 0.05). 

**Figure 6:**
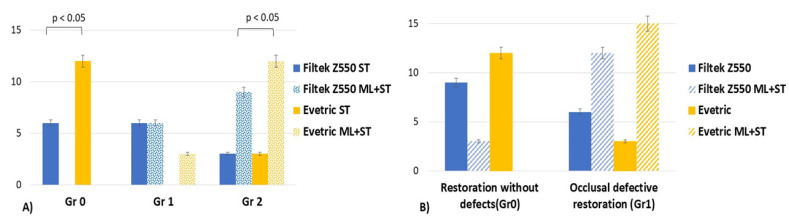
(**A**) Error bars chart representing the ‘grades’ distribution of interfacial defects for mechanically loaded (ML + ST) and non-mechanically loaded (ST) restorations of both resin-based materials. (**B**) Error bars chart representing the ‘grades’ distribution of occlusal defects for mechanically loaded (ML + ST) and non-mechanically loaded (ST) restorations of both tested materials.

For Evetric restorations, on one side, the non-mechanically simulated restorations (ST) did not present any interfacial defects compared with Filtek Z550 samples (Gr 0, *p* < 0.000001); on the other side, a statistically significant higher number of restorations graded at level 2 with interfacial defects after high-impact bruxism force simulation (ML + ST) was accomplished compared with Filtek Z550 samples (*p* = 0.009) (Figure 6). Both resin-based composite materials had statistically significant differences in Gr 2 interfacial defects after simulation of parafunctional occlusal forces (ML + ST) compared to non-mechanically loaded samples (ST) (*p* < 0.05).

When comparing by material, the Kruskal–Wallis test did not reveal any statistical difference between the tested resin composites for both mechanically and non-mechanically loaded groups no matter the presence of occlusal defects (*p* > 0.05). Regarding occlusal defects, there were statistically significant differences observed between Gr 0 and Gr1 among the samples restored with Evetric material subjected only to surface treatments (ST) (*p* = 0.009). In contrast, the samples restored with the same material subjected to high-impact parafunctional force simulation (ML + ST) showed a statistically significantly higher number of occlusal defects (Gr 1) compared with the Gr 0 mechanically simulated probes (*p* < 0.000001) (Figure 6B).

Importantly, Spearman’s rho correlation coefficient revealed a strong relationship between anatomical form parameter (AF)—(our scoring system) occlusal defects and marginal adaptation parameter (MA)—interfacial defects, respectively (ST and ML + ST groups) for both tested biocomposite restorative materials (*p* < 0.05).

## 4. Discussion

For many decades, indication of biocomposite materials for various clinical situations has increased significantly. However, these dental restorative biomaterials are subjected in the oral environment (abnormal occlusal forces, external bleaching, consumption of carbonated beverages) to changes in their functional and mechanical behavior when indicated primarily for patients with masticatory habits. 

The study aimed to recreate one of the most common situations of a young patient suffering from specific parafunctional occlusal stress (bruxism) who consumes acidic beverages and uses at-home dental bleaching. 

Dental aesthetics has always been a primary concern. Thus, home-use dental bleaching gels or other whitening pastes are now widely available online. Bleaching gels often advertise that 5 min home use is enough to whiten one’s teeth [25]. The target audience for this type of product is teenagers and young adults ages 17 to 30. It is called into question whether these types of gels, indicated by the dentist, have a mild or strong effect only on natural teeth or dental restorations. Because the bleaching agent acts on the surface of the teeth or restorations, it was observed that it may affect fracture toughness of resin-based composite restorations [30,31]] and may lead to release of some organic matrix polymers in the oral environment [32]. According to Schuster L. et al., bleaching procedures may cause reduced or enhanced elution of chemicals from the composites [33].

Another critical issue nowadays is the high percentage of patients who consume large quantities of sports drinks (Gatorade, Isostar, etc.) and carbonated beverages (e.g., Coca-Cola, Pepsi, Fanta, etc.), who represent the same target audience mentioned above [10,11]. When these two extrinsic elements are combined with intrinsic ones, such as parafunctional occlusal forces, it is essential to understand the affected dental areas and what the practitioner can use to restore the teeth [15,16,17,18,19,20]. 

In our in vitro investigation, we combined two surface treatments (home-use dental bleaching gel and immersion in sports drinks and carbonated beverages) with high-parafunctional masticatory simulation to assess both qualitatively and quantitatively the behavior of two resin-based biocomposites. Thus, we used a dual-axis chewing simulator with the loading force established at 70N [8] corresponding to the non-physiological masticatory force of a bruxer human subject [34]. It must be pointed out that there are not enough studies on this specific subject—consumption of acidic beverages and simulated bruxism forces in association with uncontrolled at-home tooth whitening procedures and their effect on mechanical behavior of composite restorative materials. 

Qualitative analysis revealed significantly rougher surfaces for Evetric biocomposite subjected to surface treatments compared to Filtek Z550. This result may be explained based on the chemical composition of the restorative materials: Evetric contains pre-polymerized particles and a distribution of 55–57% vol. of the inorganic fillers, while Filtek Z550 contains 68% vol. distribution (nanoparticles and nanoclusters). Given the values, it can be noted that the fillers protect the remaining organic matrix for Filtek Z550. Thus, it might be a higher chance for Evetric to wear its surface matrix much easier due to the low volume distribution of the filler particles [35]. (Table 2).

**Figure 7:**
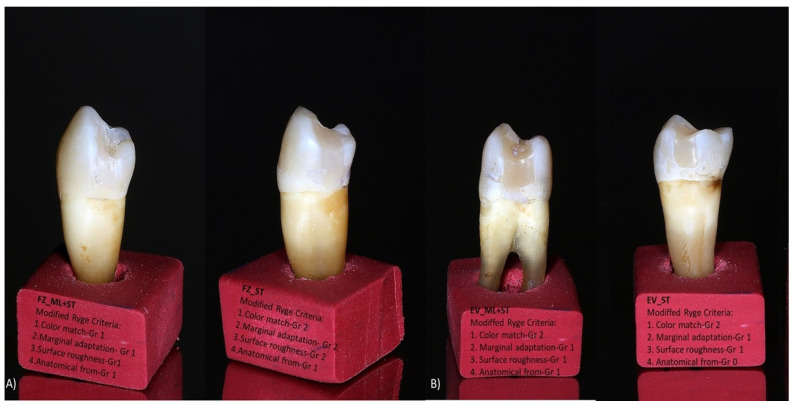
Photos of restored samples representing modified clinical parameters criteria for (**A**) Filtek Z550-ML + ST and ST groups and (**B**) Evetric ML + ST and ST groups.

The slight color change observed is based on the size of the particles mentioned above. Filtek Z550 has smaller particles and, therefore, has a smoother surface and will have lower external coloration (Figure 7); the same results were obtained by Ozkanoglu S. et al. [35]. Although nanohybrid composite resins should be resistant to external coloration, Güler et al. and other researchers [36,37] reported that excess silane-binding agent and resin amount have a significant difference that increases coloration. Therefore, similar behavior of the color parameters of grades 1 and 2 was observed for the tested samples (Figure 4 and Figure 7).

When discussing the materials’ composition, two other aspects are worth mentioning: the influence of filler percentage and the photoinitiator. Dikova T et al. [38] conducted an in vitro study that analyzed three composites (Filtek One Bulk Fill, Evetric, and FC G-aenial Universal Flo) and concluded that the Filtek material (with a similar distribution of matrix and filler particles to Filtek Z550), due to its higher filler content and composition, has the highest microhardness in comparison to Evetric [39]. Similar results were also obtained in our study: Filtek Z550 showed better preservation than Evetric of the initial anatomical form after high parafunctional simulated forces (Figure 3).

It is essential to note that properties of composite materials are also influenced by a patient’s diet (erosive factors), the materials’ composition, and the polymerization method [40,41]. 

In our study, the tested nanohybrid composites had different photoinitiators: Ivocerin (390–445 nm wavelength), for Evetric and for Filtek Z550, camphorquinone (CQ; 360–510 nm wavelength). Kowalska A. et al. [39] demonstrated that the highest hardness and microhardness are associated with Filtek Z550, which contained CQ as a photoinitiator [35]. In our study, the LED light-curing unit had a very narrow range of wavelength (440–480 nm), which increased the possibility for Evetric to have a higher depth of cure and polymerization degree, even if the filler particles content was low [38,39]; on the other side, in the case of Filtek Z550, there was higher inorganic filler content and a wider range of the wavelength compared with that of the LED lamp. Our investigation observed similar changes (*p* > 0.05) in mechanical surface behavior related to anatomical form and occlusal defects of the tested composite materials when surface treatments (ST) were simulated (Figure 4, Figure 5C, Figure 6 and Figure 7). Our findings are in accordance with other studies [8,18,38,39].

On the X-ray and CBCT images, it was observed that the samples restored with Evetric were more radio-opaque than those restored with Filtek Z550 (Figure 5A,C). This observation agrees with the results reported by other researchers [7,42,43]. Thus, the following possible explanation may be taken into consideration: within the inorganic phase composition, Evetric has different variants of filler particles (BaO glass, Ytterbium fluorides, oxides) and a low number of variants of organic polymers (Table 2) [28], while Filtek Z550 is highly represented by a variety of organic matrix monomers and only a few types of inorganic fillers (Zirconia and Silica particles) [7,43], so, the higher the variety of inorganic filler particles, the higher the radio-opacity.

Use of micro-CT is considered a “golden standard” non-destructive radiographic method for evaluating small areas for different human structures [23], especially detection of tooth-restorative material interfacial defects [22,23,24,44,45,46]. This method uses high radiation values for tiny areas and thus may be used only with a specific type of prepared samples along with laboratory investigations [23,44,45,46]. To better detect defects, dental samples are immersed in 50% silver nitrate solution [44,45,46]

For many years, cone beam computer tomography (CBCT) has been widely used for different dental specialties, especially surgical ones. This non-invasive investigation method was preferred in the past decade and is often indicated for endodontics and caries detection [21,24,47]. With CBCT, it is possible to explore dental tissues based on three-dimensional analysis, which can be completed from different plans and sectioning of the assessed tissues at different levels [21,23]. Thus, CBCT analysis on a daily dental basis may help a dental practitioner with more elements and information three-dimensionally evaluated than simple radiography at high resolution. Thus, both methods may be corroborated by the dental clinician to improve a single radiographic analysis.

The reason for using a cone beam CT device for our investigation was based on its clinical indication by dental practitioners [21,24,47]. This analysis was accomplished by respecting the conditions of clinical practice to identify its limits as correctly as possible. Our results have demonstrated similar detections with micro-CT images used by other investigators [44,45].

In our study, quantitative analysis completed with an X-ray and CBCT device showed significantly higher interfacial and occlusal defects for Evetric than Filtek Z550 (Gr 2, *p* < 0.05) after the samples were subjected to high-impact masticatory forces simulation (Figure 5 and Figure 6). Other studies also reported similar results [14,20,44].

When interfacial defects were analyzed, based on silver nitrate infiltration, CBCT images revealed statistically significant higher differences between the samples of both materials when these were subjected to bruxism simulation compared with those samples that underwent only surface treatments (immersion in carbonated juice and dental bleaching gel) (*p* < 0.05). Other researchers also observed similar effects [19,44,45,46,48]; moreover, for interfacial microleakage analysis, they reported [44,45,46] immersion of the samples in silver nitrate 50%, which is the same tracer used in our study.

Summing up, the Spearman rho correlation coefficient revealed a relationship between the two variables, which may strengthen the reliability of our grading system. Moreover, there is a correlation between the clinical parameters that evaluate the restorations exteriorly (modified clinical parameters criteria) and the internal ones (X-rays and CBCT).

The present study was based on the hypothesis that there is no difference between the tested composites regarding their mechanical behavior, aesthetics, and functional behaviors regardless of type of surface aging treatment and bruxism simulation forces. After qualitative and quantitative analysis, the null hypothesis was rejected. 

Our study had some limitations, such as laboratory setup, simulation of high parafunctional occlusal forces, type of restorative materials, and lack of elution chemical compounds collected for both assessment methods (ST and ML + ST), as well as the use of a thermocycler. Nevertheless, we want to emphasize some of the strong points of this investigation: simulation of the periodontal ligament for tooth mobility using a polyvinyl siloxane material along with the Willytec Chewing simulator type (C.S.-4.2, SD Mechatronik, Feldkirchen-Westerham, Germany); to our knowledge, this study design has not yet been reported by different groups of researchers from another academic university. Along with periodontal ligament simulation, the whole laboratory setup had the goal to implement a real clinical situation from a daily basis dental activity. The restored teeth were placed in contact proximally so that the ceramic stylus met both restored teeth. Another strong point is use of the assessment devices (high-resolution camera, digital X-rays, and CBCT device) recommended daily by dental practitioners. Moreover, to analyze the restored samples, we used loupes and digital images, enabling us to evaluate the dental restorations from an extrinsic and intrinsic 3D point of view.

However, more clinical and in vitro studies should analyze this vast domain of cone-beam computed tomography assessment and the effects of combined bruxism–acid beverages–dental bleaching on more types of dental restorative materials, other types of dental fillings, and different lab-simulated clinical setups.

## 5. Conclusions

Within the limits of this in vitro investigation, the following conclusions may be drawn:

- simulation of bruxism forces combined with surface treatments (immersion in carbonated juice and external bleaching) induced different mechanical and functional behavior patterns of the analyzed resin-based biocomposites.
- radiographic and CBCT evaluation revealed more details regarding the mechanical behavior of the tested restorative biomaterials compared with other analyzed methods (digital camera and loupes).
- use of different surface agents (carbonated juice and dental bleaching agent) dramatically impacts surface behavior of tested materials for simulated bruxism conditions.
- higher radio-opacity was observed for Evetric compared with Filtek Z550.
- more cervical defects were observed on radiographic and CBCT images for Evetric compared with Filtek Z550.
- color parameter indicated a slight color change between tested materials.
- aging treatments increased surface roughness of Evetric restorations compared with Filtek Z550 samples.

## Figures and Tables

**Table 1:** The grading system used based on modified clinical parameters criteria.

Grade	Color Match (CM)	Marginal Adaptation (MA)	Surface Roughness (SR)	Anatomical Form (AF)
GR 0	No change in color	The tooth-restoration margin is undetectable.	Smooth surface of the restoration	No change of the ana-tomical shape
GR 1	Slight change in color	Detectable tooth-restoration margins without any crevicular ditch	Slightly rough surface of the restoration	Detectible under- or over-contoured ana-tomical shape of the restoration
GR 2	Obvious change in color of the restoration	Detectable tooth-restoration margins with visible crevicular space	Rough surface of the restoration	

**Table 2:** Chemical composition of the restorative materials used in the study [7,28].

Material [Lot Number]	Organic Matrix (+ Photo-Initiator) *	Inorganic Phase *	Medium Particle Size *	Particles’ Distribution wt% (vol%) *	Manufacturer
Evetric (EV) [Z03XB7] A3 shade	-BisGMA -BisEMA -UDMA +Ivocerin^®^	-Barium glass -Ytterbium trifluoride -Oxides -Pre-polymerized particles	0.04–3 µm	80–81 (55–57)	Ivoclar, Vivadent, Schaan Liechtenstein
Filtek Z550 (FZ) [NC35371] A3 shade	-BisGMA -BisEMA -UDMA -PEGDMA -TEGDMA +Camphorquinone (CQ)	-Zirconium oxide silica -Silica particles	0.005–3 µm (Cluster 0.6–1.4 µm)	82 (68)	3M ESPE, St Paul, MN, USA

BisGMA: bisphenol A diglycidyl ether dimethacrylate; TEGDMA: triethylene-glycol dimethacrylate BisEMA6: bisphenol A polyethylene glycol diether dimethacrylate; UDMA: urethane dimethacrylate; PEGDMA: polyethylene glycol dimethacrylate; * in accordance with the information provided by the manufacturers.

**Table 3:** The chemical composition of the adhesive used in the study [29].

Material	Composition *	Manufacturer
Optibond eXTRa Universal two-step self-etch Adhesive [Primer: 7247705 Adhesive:7247706]	Primer: GPDM, hydrophilic co-monomers, water/ethanol, acetone Adhesive: resin monomers, inorganic fillers, ethanol	Kerr Corporation

* In accordance with the information provided by the manufacturers.

**Table 4:** Kruskal–Wallis test for color match parameter (CM).

Test Statistics ^a,b^				
	CM_ Filtek Z550 ST	CM_ Evetric ST	CM_ Filtek Z550 ML + ST	CM_ Evetric ML + ST
Chi-Square	22.880	35.787	9.387	12.320
df	2	2	2	2
Asymp. Sig.	0.000	0.000	0.009	0.002

a. Kruskal–Wallis test; b. grouping variable: grade CM.

**Table 5:** Kruskal–Wallis test for surface roughness (SR) parameter.

Test Statistics ^a,b^				
	SR_ Filtek Z550 ST	SR_ Evetric ST	SR_ Filtek Z550 ML + ST	SR_ Evetric ML + ST
Chi-Square	4.808	15.840	2.347	4.107
df	2	2	2	2
Asymp. Sig.	0.090	0.000	0.309	0.128

a. Kruskal–Wallis test; b. grouping variable: grade SR.

**Table 6:** The grading system used in the study to assess occlusal defects of dental restorations.

Grade	Significance
0	-without occlusal defects
1	-occlusal defects

**Table 7:** The grading system used in the study to assess interfacial defects of dental restorations.

Grade	Significance
0	-without interfacial defects
1	-interfacial defects at the cervical
2	-interfacial defects at the cervical level and pulpal wall

## Data Availability

The data supporting this study’s findings are available on request from the corresponding author.
